# The 28 + 28 day design is an effective sampling time for analyzing mutant frequencies in rapidly proliferating tissues of MutaMouse animals

**DOI:** 10.1007/s00204-021-02977-6

**Published:** 2021-01-28

**Authors:** Francesco Marchetti, Gu Zhou, Danielle LeBlanc, Paul A. White, Andrew Williams, Carole L. Yauk, George R. Douglas

**Affiliations:** 1 Environmental Health Science and Research Bureau, Healthy Environments and Consumer Safety Branch, Health Canada, 251 Sir Frederick Banting Driveway, Ottawa, ON K1A 0K9 Canada; 2 Department of Biology, University of Ottawa, Ottawa, ON Canada

**Keywords:** Benzo[a]pyrene, Isopropyl methanesulfonate, Procarbazine, Triethylenemelamine, Benchmark dose

## Abstract

The Organisation for Economic Co-Operation and Development Test Guideline 488 (TG 488) uses transgenic rodent models to generate in vivo mutagenesis data for regulatory submission. The recommended design in TG 488, 28 consecutive daily exposures with tissue sampling three days later (28 + 3d), is optimized for rapidly proliferating tissues such as bone marrow (BM). A sampling time of 28 days (28 + 28d) is considered more appropriate for slowly proliferating tissues (e.g., liver) and male germ cells. We evaluated the impact of the sampling time on mutant frequencies (MF) in the BM of MutaMouse males exposed for 28 days to benzo[a]pyrene (BaP), procarbazine *(*PRC), isopropyl methanesulfonate (iPMS), or triethylenemelamine (TEM) in dose–response studies. BM samples were collected + 3d, + 28d, + 42d or + 70d post exposure and MF quantified using the *lacZ* assay. All chemicals significantly increased MF with maximum fold increases at 28 + 3d of 162.9, 6.6, 4.7 and 2.8 for BaP, PRC, iPMS and TEM, respectively. MF were relatively stable over the time period investigated, although they were significantly increased only at 28 + 3d and 28 + 28d for TEM. Benchmark dose (BMD) modelling generated overlapping BMD confidence intervals among the four sampling times for each chemical. These results demonstrate that the sampling time does not affect the detection of mutations for strong mutagens. However, for mutagens that produce small increases in MF, sampling times greater than 28 days may produce false-negative results. Thus, the 28 + 28d protocol represents a unifying protocol for simultaneously assessing mutations in rapidly and slowly proliferating somatic tissues and male germ cells.

## Introduction

Transgenic rodent (TGR) mutation models currently represent the most effective tool to evaluate the ability of chemicals to induce mutations in vivo (Lambert et al. 2005; OECD 2009). The use of TGR models to generate in vivo mutagenicity data for regulatory decision-making is described in the Organisation for Economic Co-Operation and Development (OECD) test guideline (TG) 488 that has been recently updated (OECD 2020). Unlike some of the other existing in vivo TGs for genotoxicity that measure the response in only one tissue, e.g., the in vivo micronucleus (MN) assay (OECD 2016), TGR models allow the analysis of mutations in virtually any tissue (Gingerich et al. 2014). A noteworthy exception is female germ cells since it is not possible to collect a sufficient number of eggs to obtain enough DNA for conducting the TGR assay (Yauk et al. 2005). While the majority of TGR models detect mostly point mutations and short indels (Lambert et al. 2005), the TGR assay can be easily integrated with the MN assay and other genotoxic endpoints (Hori et al. 2019; Lemieux et al. 2011; Long et al. 2018; Maurice et al. 2019) for the simultaneous analysis of both mutations and chromosomal events.

Germ cell mutagenicity assessment has become increasingly critical because of the wide implementation of the Globally Harmonized System (GHS) of classification and labelling of chemicals (United Nations 2017) that requires the assessment of germ cell hazard for proper chemical classification. The TGR assay is currently the only OECD TG that evaluates the same endpoint in both somatic tissues and male germ cells. However, specification of a study design that supports mutagenicity assessment of both somatic tissues and germ cells is challenging due to the unique characteristics of spermatogenesis (e.g., distinct germ cell types, lengthy duration, etc.).

In the mouse, it takes about 49 days to generate mature sperm from spermatogonial stem cells (Marchetti et al. 2018a). During this time, active proliferation is restricted to the spermatogonia compartment and for the last 35 days of male germ cell development there is no DNA replication. The respective times in the rat are approximately 70 and 50 days (Marchetti et al. 2018a). The role of spermatogonial stem cells in maintaining the production of mature male germ cells is well documented and understood (de Rooij 2001). In contrast, the role of hematopoietic stem cells in generating fully developed hematopoietic cells is still unclear, with mounting evidence that hematopoiesis in the adult is sustained by lineage-specific multipotent progenitor cells (Busch et al. 2015; Rodriguez-Fraticelli et al. 2018; Sun et al. 2014). Compared to spermatogenesis, the time necessary to generate mature hematopoietic cells is considerably shorter (Abramsson-Zetterberg et al. 1996); moreover, hematopoietic cells are replicating with an average doubling time of about 10 h (Pacchierotti et al. 1991). These differences in replicative capacity and cell cycle duration are particularly important because DNA replication and cell division are essential to convert transient DNA damage into a permanent and measurable mutation (Bielas and Heddle 2000), Thus, an experimental design that is adequate for the detection of induced mutations in the bone marrow (BM) may not be appropriate for germ cells, and vice versa.

Two experimental variables that impact the characterization of the mutagenic properties of a chemical are the treatment duration and the elapsed time from the last exposure to tissue collection for analysis (i.e., sampling time). Daily exposures over a period of four weeks are considered a good compromise to allow sufficient accumulation of mutations in slowly proliferating tissues (Heddle et al. 2000) and limit the chance of false positives due to clonal expansion in rapidly proliferating tissues (Thybaud et al. 2003). The sampling time impacts the magnitude of the measured response depending on the rate of proliferation and turnover of the tissue (Heddle et al. 2003), with rapidly proliferating tissues expected to reach the maximum mutant frequency (MF) earlier than slowly proliferating tissues (Thybaud et al. 2003). To maximize the chance of detecting an effect, both exposure time and sampling time should be specific to each tissue. However, such an approach would be impractical because it would require a different set of animals for each tissue and a compromise is necessary to effectively assess responses in both rapidly and slowly proliferating tissues.

The TGR assay is commonly conducted by exposing animals for 28 consecutive days and collecting tissues three days after the last exposure (i.e., 28 + 3d study design). This sampling time was selected based on data demonstrating that MF reached a peak in BM three days after an acute exposure (Thybaud et al. 2003) and to reduce the chances of ex vivo mutations originating from unrepaired DNA damage present in the transgene at the time of tissue collection (Heddle et al. 2003). While TG 488 acknowledges that longer sampling times (e.g., the 28 + 28d design) are more appropriate for slowly proliferating tissues, such as the liver, the assay is most commonly conducted using the 28 + 3d design because of concerns about the impact of extending the sampling time on the detection of mutations in rapidly proliferating tissues. A few cases of an apparent decline in MF observed in BM with increased sampling times have been reported (Heddle et al. 2003; Thybaud et al. 2003). However, some of these results were obtained using a single acute exposure (Hara et al. 1999), which may not be representative of outcomes following a 4 week exposure. Other reported MF declines were not statistically significant (Thybaud et al. 2003). Thus, we contend that there are currently insufficient data to evaluate the full impact of extending the sampling time (e.g., the 28 + 28d design) for the analysis of MF in rapidly proliferating tissues.

A major disadvantage of the 28 + 3d design is the fact that this regimen is ineffective for assessing germ cell mutagenicity. Evaluation of TGR germ cell data obtained with the 28 + 3d design showed that measurement of MF in both caudal sperm and germ cells from the seminiferous tubules is prone to false negatives (Marchetti et al. 2018b; O'Brien et al. 2016). This is because a three-day sampling time does not provide sufficient time for germ cells exposed during the proliferating phase of spermatogenesis, when mutations in the transgene can be induced, to populate the testis (Marchetti et al. 2018a). Conversely, modeling of spermatogenesis showed that the 28 + 28d design significantly improves the exposure sustained during the proliferating phase of spermatogenesis (Marchetti et al. 2018a). Together with empirical data [reviewed in (Marchetti et al. 2018b)], this model formed the basis for the recent revision of TG 488, which recommends the 28 + 28d design for assessment of germ cell mutagenicity (OECD 2020).

The revision of TG 488 pertaining to germ cell study designs further proposes the 28 + 28d design as a common protocol for simultaneously assessing mutagenicity in somatic tissues and germ cells of the same animals. However, additional data are urgently needed to evaluate the impact of the 28 + 28d design on the detection of mutations in rapidly proliferating tissues. Here, we investigated the influence of the sampling time on MF in BM (i.e., a rapidly proliferating tissue) of MutaMouse animals exposed to four established mutagens with greatly different mutagenic responses: benzo(a)pyrene (BaP) (Lemieux et al. 2011); procarbazine (PRC) (Maurice et al. 2019; Suzuki et al. 1999), isopropyl methanesulfonate (iPMS) (Coffing et al. 2015; Itoh et al. 2016); and triethylenemelamine (TEM) (Maurice et al. 2018). Each chemical was tested using a 28 days repeated-dose treatment and at least three doses alongside concurrent controls. BM was collected on post-exposure days + 3, + 28, + 42 or + 70. MF were determined using the *lacZ* assay and the Benchmark Dose (BMD) approach used to evaluate the effect of sampling times.

## Materials and methods

### Animals

The use of mice in these studies was approved by the Health Canada Ottawa Animal Care Committee. All animal exposures and handling were conducted in accordance with the Guidelines of the Canadian Council on Animal Care (CCAC) described in the CCAC Guide to the Care and Use of Experimental Animals (CCAC 1993). MutaMouse males for these experiments were generated from an in-house colony of animals each harboring ~ 29 tandem copies of a recombinant λgt10 phage shuttle vector on each copy of chromosome 3 (Shwed et al. 2010). Mice were maintained under a 12 h light/ 12 h dark photoperiod at room temperature of 21 °C and relative humidity of 50% with access to water and food ad libitum throughout the acclimation and experimental periods. Adult MutaMouse males, 9–14 weeks of age at the beginning of the exposure, were randomly assigned to dose groups; typically, four mice for the control group, and eight mice per treatment group per time point. Animals were individually housed in microVENT ventilated racks (Allentown, Allentown, NJ).

### Chemicals and exposures

BaP (CAS 50–32-8), PRC (CAS 366–70-1) and iPMS (CAS 926–06-7) were purchased from Sigma-Aldrich Canada Co. (Oakville ON Canada). TEM (CAS 51–18-3) was bought from Synchem Ug and Co.KG (Felsberg, Altenburg, Germany). BaP and iPMS were dissolved in olive oil (Sigma-Aldrich, Oakville ON Canada), while PRC and TEM were prepared with phosphate-buffered saline without calcium and magnesium (PBS; Corning cellgro, Manassas, VA, USA).

For each chemical, the top dose was selected based on pilot dose range-finding studies to exclude doses that induced excessive morbidity or toxicity, such as approaching a 20% decrease in body weight (bw). Dose levels for the main studies were: 0, 12.5, 25, 50 and 100 mg/kg bw for BaP; 0, 6.25, 12.5 and 25 mg/kg bw for PRC; 0, 1.25, 2.5 and 5 mg/kg bw for iPMS; and 0, 1, 2, and 5 mg/kg bw for TEM. Dissolved chemicals and vehicle controls were administered daily by oral gavage in a volume of 5 mL/kg bw for 28 consecutive days according to TG 488. Animals were euthanized by cardiac puncture under isoflurane anesthesia at + 3d, + 28d, + 42d or + 70d post exposure. The femurs were removed and BM was flushed out in 1 mL of PBS and centrifuged at 10,000 rpm for 30 s; pellets were resuspended in 100 μL of PBS, and frozen in liquid nitrogen and stored at −  80 °C until use.

The four sampling times for BaP represent three experiments that were conducted at different times. The first experiment included doses up to 50 mg/kg BaP and did not include the + 28d sampling time; the second experiment used only the 100 mg/kg BaP dose and, as in the first one, did not include the + 28d sampling time; and, the third experiment included all doses with just the + 28d sampling time. Each experiment had its own concurrent controls. Conversely, the four sampling times for PRC, iPMS and TEM were conducted as parts of single experiments where all animals were treated at the same time and randomly allocated to one of the four sampling times. In these experiments, only four control animals were used at each sampling time in an effort to adhere to 3Rs principles, and with the intention of combining all controls at the four sampling times into a single group for statistical analyses.

### DNA extraction

Total genomic DNA from BM samples was extracted as previously described (Gingerich et al. 2014). Briefly, 50 μL of thawed BM was added into 5 mL lysis buffer [10 mM EDTA, 100 mM NaCl, 10 mM Tris–HCl, pH 7.6, 1% sodium dodecyl sulfate (w/v) and 1 mg/mL Proteinase K (Invitrogen, Burlington, Canada)], and digested at 37 °C overnight with gentle shaking. Genomic DNA was isolated using the phenol/chloroform extraction procedure. Precipitated DNA was dissolved in 30–75 μL TE buffer (10 mM Tris pH 7.6, 0.1 mM EDTA) and stored at 4 °C. The quantity and quality of DNA were assessed using a NanoDrop spectrophotometer (Thermo Scientific Canada, Ottawa, Canada).

### Mutant frequency analysis

BM samples used in these studies are from the same animals whose MF were previously reported for PRC (Maurice et al. 2019), TEM (Maurice et al. 2018), and BaP (Beal et al. 2015). However, except for the high dose of BaP at 28 + 3d, where no DNA was available, new DNA isolations and *lacZ* assays were conducted to generate the MF data reported here.

λgt10 phage vectors in genomic DNA were excised using commercial packaging extract kits (Agilent technology, Santa Clara, CA, USA) according to the manufacturer’s instructions. Transgene MF was determined using the phenyl *β*-d-galactopyranoside (P-gal)–positive selection assay (Lambert et al. 2005; Vijg and Douglas 1996). Briefly, packaged phage particles were adsorbed to E. coli host cells (lacZ^−^/galE^−^); the host cells were mixed with selective LB top agar containing 0.3% phenyl *β*-d-galactoside (P-gal; Sigma-Aldrich, Oakville ON Canada) and then plated. Concurrently, a small proportion of bacteria with adsorbed phage particles were mixed with nonselective LB top agar to estimate total plaque-forming units (pfu). All plates were incubated overnight at 37 °C. A minimum of 125,000 total pfu were scored for each animal. MF was expressed as the ratio of mutant plaques to total pfu.

### Statistical analyses

Except in a few cases, multiple *lacZ* packaging reactions were needed to reach the minimum number of pfu for each animal. Thus, to identify outlier replicates, we first analyzed MF from each reaction using a generalized linear mixed model (GLMM) with a binomial error distribution in R software. This model accounts for the dose effect, and also for random differences between individuals. Observations were identified as outliers whenever the model residuals were greater than a cut-off point; typically, > 3 standard deviations of the mean (Gelman and Hill 2007). The most extreme outliers were removed, and the analysis repeated until all outlier replicates were removed. In total, 18 replicates out of 960 (1.8%) were removed. The data were then collapsed by animal and analyses conducted again to identify individual animal outliers. This resulted in the elimination of three animals out of 461 (0.65%). All three animals were controls in the BaP experiment. Estimated MF were obtained using generalized linear models in the R software with the quasibinomial distribution. Pairwise comparisons were conducted using the doBy R library (Højsgaard and Halekoh 2018) and these estimates were then back transformed. Here, the delta method was employed to approximate the back-transformed standard errors of the estimated MF. Finally, a Holm-Sidak correction was applied to adjust p-values for multiple comparisons.

### Dose–response modelling and benchmark dose (BMD) determination

The BMD combined-covariate approach was employed to examine the influence of sampling time on the potency (i.e., BMD) of each tested chemical (Wills et al. 2016). BMD analyses were conducted using PROAST in R (version 67.1, https://rivm.nl/en/proast). Following the recommendation of White et al. (2020), the benchmark response (BMR) was set to 50%. To ensure consistency of the analyses across the tested substances, the dose–response data were analysed using a five parameter exponential model. BMD-covariate analyses often assume that some model parameters (e.g., *c* and *d* for maximum response and log-steepness after axis scaling) are constant across covariate sub-groups (Wills et al. 2017); this is consistent with the work of (Slob and Setzer 2014). The AIC (Akaike Information Criterion) was used for model selection, and post-hoc analyses statistically evaluated the effect of sampling time on any retained parameter. The upper and lower confidence limits of the BMD values (i.e., the BMDL and BMDU) were used to statistically evaluate the effect of sampling time.

## Results

### Analyses of mutant frequencies in control animals by age

**Fig. 1:**
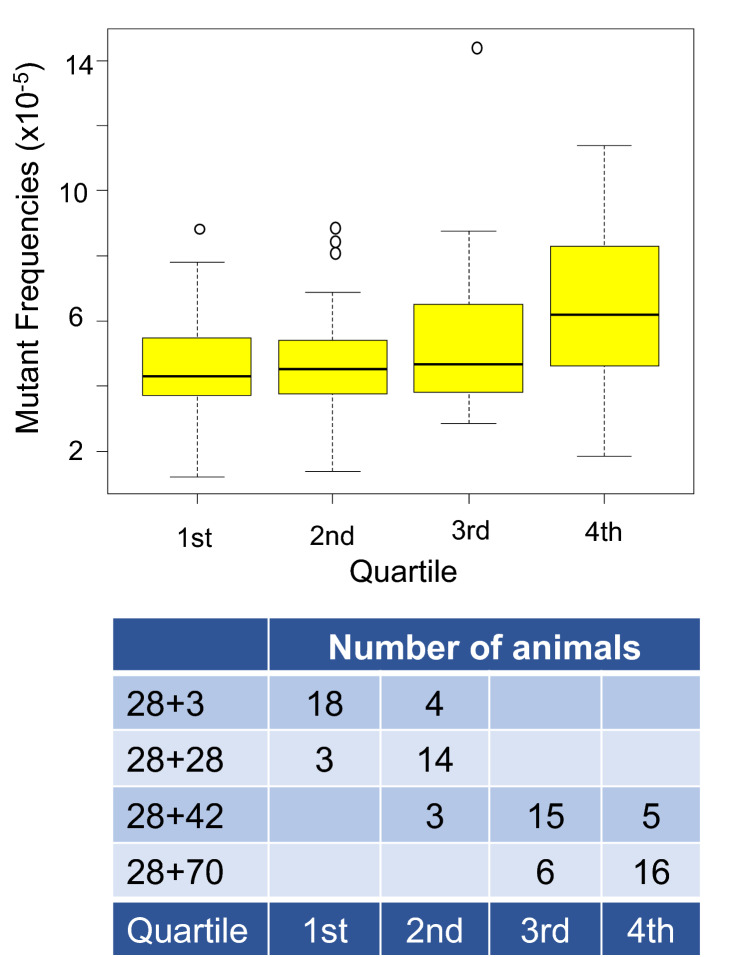
Mutant frequencies in control animals by age. The top panel shows the median mutant frequencies in control animals by age quartile. Median mutant frequencies were 4.3, 4.5, 4.7 and 6.2 × 10^–5^ for 1st, 2nd, 3rd, and 4th quartile, respectively. There is a borderline significant trend (*p* = 0.06) for an age effect. The bottom panel shows the distribution of control animals from the four sampling times among quartiles

A total of 84 control animals were used in these studies. Table 1 shows the *lacZ* MF by sampling time across the four chemical experiments for the control mice. An average of 339,608 ± 171,139 (mean ± SD) pfu per control animal was analyzed. We found that MF (× 10^–5^) tended to increase with sampling time, with MF (± standard deviation) of 4.3 ± 1.9, 5.4 ± 1.7, 5.8 ± 2.8 and 5.7 ± 2.3 at 28 + 3d, 28 + 28d, 28 + 42d and 28 + 70d, respectively. Furthermore, the MF at 28 + 42d was significantly higher (*p* < 0.03) than that observed at 28 + 3d. A longer sampling time necessarily results in older animals; however, the age of the animals at the beginning of each experiment also varied. Therefore, rather than using the sampling time as a proxy for chronological age, we also considered the litter’s day of birth with respect to euthanasia to better analyze the association between MF and age. Because we could not separate the animals into four groups with no age overlap based on the day of birth, MF were analyzed by quartile. This analysis showed a clearer age effect with MF increasing by quartile (Fig. 1). In fact, median MF × 10^–5^ were 4.3, 4.5, 4.7 and 6.2 for the 1st, 2nd, 3rd and 4th quartile, respectively. The difference between the 1st and 4th quartile was at the borderline of significance (*p* = 0.06). Figure 1 also shows that controls from 28 + 3d and 28 + 28d represented 39 of the 42 animals in the first two quartiles, and controls from 28 + 42d and 28 + 70d represented all of the animals in the last two quartiles.

**Table 1:** *LacZ* mutant frequencies in the bone marrow of MutaMouse control mice by sampling time

Sampling time (days)	No. of animals	No. of Mutants	No. of pfu^a^	Average^b^ MF × 10^–5^	SD^b^
28 + 3	22	372	8,254,339	4.3	1.9
28 + 28	17	240	4,444,173	5.4	1.7
28 + 42	23	436	7,770,245	5.8*	2.8
28 + 70	22	434	8,058,374	5.7	2.3

^a^Plaque forming units (pfu)

^b^Average mutant frequency (MF) and standard deviation (SD) of the arithmetic mean of individual animals

^*^p < 0.03 vs 28 + 3d sampling time

These results did not support the merging of the controls from all four sampling times within each experiment, as it was originally planned in an effort to reduce the number of animals used and adhere to the 3Rs principles. Instead, in each experiment, we merged controls at 28 + 3d and 28 + 28d to analyze the chemical response at these two timepoints and merged controls at 28 + 42d and 28 + 70d to analyze the chemical response at the two later timepoints.

### Qualitative analyses: effect of the sampling time on mutant frequencies in bone marrow

**Fig. 2:**
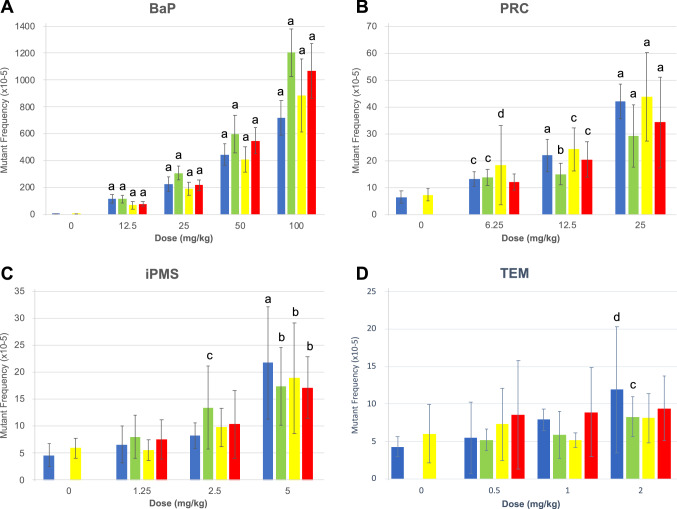
Mutant frequencies in the bone marrow of MutaMouse males at the various sampling times. The frequencies of *lacZ* mutants in the bone marrow of MutaMouse males exposed to benzo(a)pyrene **a**, procarbazine **b**, isopropyl methanesulfonate **c** and triethylenemelamine **d** were evaluated at 28 + 3d (blue), 28 + 28d (green), 28 + 42d (yellow) and 28 + 70d (red). For each dose group, the mean ± standard deviation is shown. In each experiment, controls at 28 + 3d and 28 + 28d were merged into a single control group (blue bar) that was used to analyze the dose–response at these two time points. Similarly, controls at 28 + 42d and 28 + 70d were merged into a single control group (yellow bar) that was used to analyze the dose–response at these two time points. Note that each panel uses a different Y axis and that control bars for the BaP experiment are difficult to see because of the magnitude of the response at the high dose. Statistical results with respect to concurrent controls are presented for each dose and sampling time. The level of significance is indicated as follows: *a* = *p* < 0.0001; *b* = *p* < 0.001; *c* = *p* < 0.01; and, *d* = *p* < 0.05 (color figure online)

The *lacZ* MF measured at the four sampling times in the BM of MutaMouse males exposed to BaP are shown in Table 2 and graphically in Fig. 2a. BaP induced statistically significant dose-dependent increases in MF that reached a maximum of 1204.1 × 10^–5^ for the high dose group of 100 mg/kg bw/day at 28 + 28d. Fold changes for the BaP high dose group were between 163 and 274 depending on sampling time, while at the low dose of 12.5 mg/kg bw/day they were between 14 and 27. Regardless of sampling time, all four doses of BaP induced statistically significant increases in MF with respect to concurrent controls (*p* < 0.0001). Thus, analyses of MF in BM after BaP exposure produced qualitatively similar results at all sampling times investigated for all four doses tested.

**Table 2:** *LacZ* mutant frequencies in the bone marrow of MutaMouse mice exposed to benzo(a)pyrene and collected at various sampling times

Sampling time (days)	Dose (mg/kg/day)	No. of animals	No. of Mutants	No. of pfu^a^	Average^b^ MF × 10^–5^	SD^b^	*p* value^c^
28 + 3^d^	0	18	278	6,353,978	4.4	3.1	–
	12.5	4	1,193	1,061,591	116.7	30.1	**1.5E**–**11**
	25	6	3,291	1,481,392	226.4	55.6	**1.2E**–**15**
	50	6	5,400	1,238,524	445.8	82.5	**< 1.0E**–**16**
	100	6	18,261	2,453,689	716.7	128.2	**< 1.0E**–**16**
28 + 28^d^	0	18	278	6,353,978	4.4	3.1	–
	12.5	8	1,830	1,583,440	114.4	29.3	**1.3E**–**13**
	25	8	4,995	1,622,704	308.8	54.0	**< 1.0E**–**16**
	50	8	8,663	1,445,276	598.2	139.0	**< 1.0E**–**16**
	100	6	13,354	1,088,927	1204.1	177.1	**< 1.0E**–**16**
28 + 42^e^	0	21	451	8,433,262	5.1	2.0	–
	12.5	6	816	1,218,973	68.2	30.4	**5.0E**–**7**
	25	6	2,321	1,160,993	191.5	49.1	**2.0E**–**12**
	50	6	4,452	1,101,187	408.8	93.3	**1.8E**–**13**
	100	6	18,848	2,308,731	885.8	272.1	**< 1.0E**–**16**
28 + 70^e^	0	21	451	8,433,262	5.1	2.0	–
	12.5	5	2,330	2,968,083	78.9	17.1	**5.2E**–**11**
	25	6	5,978	2,831,739	218.0	40.9	**8.9E**–**16**
	50	5	11,755	2,223,579	547.8	96.7	**< 1.0E**–**16**
	100	6	18,140	1,798,146	1066.3	204.1	**< 1.0E**–**16**

^a^Plaque forming units (pfu)

^b^Average mutant frequency (MF) and standard deviation (SD) of the arithmetic mean of individual animals

^c^vs controls (Bold *p* values indicate statistical significance after Holm-Sidak correction for multiple comparisons)

^d^Combined controls from 28 + 3d and 28 + 28d sampling times

^e^Combined controls from 28 + 42d and 28 + 70d sampling times

The *lacZ* MF measured at the four sampling times in the BM of MutaMouse males exposed to PRC are shown in Table 3 and graphically in Fig. 2b. As for BaP, PRC induced statistically significant dose-dependent increases in MF that reached a maximum of 42.0 × 10^–5^ for the high dose group of 25 mg/kg bw/day at 28 + 3d. Fold changes for the high dose groups were between 4.5 and 6.5 depending on sampling time, while at the low dose of 6.25 mg/kg bw/day they were between 1.7 and 2.5. For PRC, the high and middle dose group (12.5 mg/kg bw/day) induced statistically significant increases in MF with respect to concurrent controls (*p* < 0.0001) at all four sampling times. For the low dose group, a statistically significant increase in MF with respect to concurrent controls was observed at all sampling times except 28 + 70d (*p* = 0.34). Except for this last result, analyses of MF in BM after PRC exposure produced qualitatively similar results at all sampling times investigated.

**Table 3:** *LacZ* mutant frequencies in the bone marrow of MutaMouse mice exposed to procarbazine and collected at various sampling times

Sampling time (days)	Dose (mg/kg/day)	No. of animals	No. of Mutants	No. of pfu^a^	Average^b^ MF × 10^–5^	SD^b^	*p* value^c^
28 + 3^d^	0	7	140	2,164,269	6.4	2.3	–
	6.25	8	380	2,909,145	13.2	2.7	**0.0034**
	12.5	8	673	3,108,736	21.9	6.0	**1.1E**–**6**
	25	8	1465	3,467,239	42.0	6.5	**7.0E**–**11**
28 + 28^d^	0	7	140	2,164,269	6.4	2.3	–
	6.25	8	188	1,384,324	13.7	3.1	**0.0052**
	12.5	8	241	1,544,345	15.0	4.0	**0.0005**
	25	7	385	1,420,095	29.1	11.6	**1.2E**–**7**
28 + 42^e^	0	8	107	1,518,994	7.2	2.3	–
	6.25	8	298	1,427,597	18.3	14.8	**0.0226**
	12.5	7	336	1,414,285	24.3	8.0	**0.0087**
	25	7	669	1,501,046	43.8	16.3	**4.0E**–**5**
28 + 70^e^	0	8	107	1,518,994	7.2	2.3	–
	6.25	7	153	1,283,787	12.1	3.0	0.3385
	12.5	7	254	1,268,714	20.4	6.6	**0.0064**
	25	8	538	1,649,728	34.2	16.8	**3.1E**–**5**

^a^Plaque forming units (pfu)

^b^Average mutant frequency (MF) and standard deviation (SD) of the arithmetic mean of individual animals

^c^vs controls (Bold *p* values indicate statistical significance after Holm-Sidak correction for multiple comparisons)

^d^Combined controls from 28 + 3d and 28 + 28d sampling times

^e^Combined controls from 28 + 42d and 28 + 70d sampling times

The *lacZ* MF measured at the four sampling times in the BM of MutaMouse males exposed to iPMS are shown in Table 4 and graphically in Fig. 2c. iPMS induced a consistent statistically significant (*p* < 0.001) increase in MF across sampling times, but only for the high dose group of 5 mg/kg bw/day. Fold-changes for the high dose group were between 4.7 and 2.9 depending on sampling time, with the MF reaching a maximum of 21.8 × 10^–5^ at 28 + 3d. The low dose of 1.25 mg/kg bw/day did not statistically increase MF with respect to controls at any of the sampling times investigated, while the middle dose of 2.5 mg/kg bw/day produced a statistically significant increase in MF of 13.4 × 10^–5^ only at 28 + 28d. Overall, the four sampling times produced qualitatively similar results.

**Table 4:** *LacZ* mutant frequencies in the bone marrow of MutaMouse mice exposed to isopropyl methanesulfonate and collected at various sampling times

Sampling time (days)	Dose (mg/kg/day)	No. of animals	No. of Mutants	No. of pfu^a^	Average^b^ MF × 10^–5^	SD^b^	*p* value^c^
28 + 3^d^	0	8	115	2,308,895	4.6	2.1	–
	1.25	6	107	1,603,984	6.6	3.4	0.7581
	2.5	8	172	2,035,545	8.3	2.3	0.2247
	5	8	457	2,131,302	21.8	10.4	**1.2E**–**5**
28 + 28^d^	0	8	115	2,308,895	4.6	2.1	–
	1.25	8	108	1,408,166	8.0	3.9	0.4028
	2.5	6	176	1,261,221	13.4	7.7	**0.0019**
	5	8	267	1,513,531	17.3	7.2	**0.0001**
28 + 42^e^	0	8	90	1,453,394	5.9	1.9	–
	1.25	8	77	1,362,941	5.6	2.0	0.9892
	2.5	8	148	1,508,725	9.8	3.5	0.2955
	5	8	340	1,591,724	18.9	10.2	**0.0001**
28 + 70^e^	0	8	90	1,453,394	5.9	1.9	–
	1.25	8	149	2,071,328	7.5	3.6	0.9424
	2.5	8	238	1,987,337	10.4	6.3	0.0648
	5	8	402	2,158,305	17.2	5.7	**0.0006**

^a^Plaque forming units (pfu)

^b^Average mutant frequency (MF) and standard deviation (SD) of the arithmetic mean of individual animals

^c^vs controls (Bold *P*-values indicate statistical significance after Holm-Sidak correction for multiple comparisons)

^d^Combined controls from 28 + 3d and 28 + 28d sampling times

^e^Combined controls from 28 + 42d and 28 + 70d sampling times

The *lacZ* MF measured at the four sampling times in the BM of MutaMouse males exposed to TEM are shown in Table 5 and graphically in Fig. 2d. Based on previous results (Maurice et al. 2018), TEM was expected to induce the lowest fold-increase in MF among the four mutagens investigated. Indeed, only the high dose of 5 mg/kg bw/day induced a statistically significant (*p* < 0.05) increase in MF at the first two sampling times, while no statistically significant increase was seen at the later two sampling times. Fold-changes at 28 + 3d and 28 + 28d were 2.8 and 1.9, respectively. Furthermore, the middle and low TEM doses did not significantly increase MF at any of the four sampling times investigated. Thus, the results with TEM suggest that the response at the first two sampling times was qualitatively different than that observed at the two later sampling times.

**Table 5:** *LacZ* mutant frequencies in the bone marrow of MutaMouse mice exposed to triethylenemelamine and collected at various sampling times

Sampling time (days)	Dose (mg/kg/day)	No. of animals	No. of Mutants	No. of pfu^a^	Average^b^ MF × 10^–5^	SD^b^	*p* value^c^
28 + 3^d^	0	6	79	1,871,370	4.3	1.3	–
	0.5	8	133	2,314,695	5.5	4.8	0.8400
	1	6	151	1,947,742	7.9	1.4	0.3656
	2	7	293	2,425,691	11.9	8.4	**0.0246**
28 + 28^d^	0	6	79	1,871,370	4.3	1.3	–
	0.5	8	139	2,669,883	5.2	1.4	0.7364
	1	8	119	1,957,186	5.9	3.2	0.3337
	2	8	180	2,036,376	8.3	2.6	**0.0059**
28 + 42^e^	0	8	249	4,422,969	6.0	3.9	–
	0.5	8	262	3,847,940	7.3	4.8	0.8592
	1	8	232	4,329,862	5.2	1.0	0.9971
	2	8	353	4,219,862	8.1	3.3	0.3230
28 + 70^e^	0	8	249	4,422,969	6.0	3.9	–
	0.5	8	256	3,831,039	8.6	7.2	0.9454
	1	8	277	3,102,774	8.9	5.9	0.4502
	2	8	411	4,558,813	9.4	4.3	0.3609

^a^Plaque forming units (pfu)

^b^Average mutant frequency (MF) and standard deviation (SD) of the arithmetic mean of individual animals

^c^vs controls (Bold *p* values indicate statistical significance after Holm-Sidak correction for multiple comparisons)

^d^Combined controls from 28 + 3d and 28 + 28d sampling times

^e^Combined controls from 28 + 42d and 28 + 70d sampling times

As summarized in Table 6, the statistical analyses just described indicated that the sampling time had little impact on the qualitative analyses of the overall response for the three chemicals that elicited strong responses. However, the results with TEM suggest that when the mutagenic response is weak, sampling times greater than 28 days may reduce the chance of detecting a significant effect.

**Table 6:** Summary of doses with statistically significant increases with respect to concurrent controls by sampling time

Chemical	Sampling time			
	28 + 3d	28 + 28d	28 + 42d	28 + 70d
Benzo(a)pyrene	L, M, MH, H	L, M, MH, H	L, M, MH, H	L, M, MH, H
Procarbazine	L, M, H	L, M, H	L, M, H	M, H
Isopropyl methanesulfonate	H	M, H	H	H
Triethylenemelamine	H	H	–	–

*L* low, *M* medium, *MH* medium high, *H* high

### Quantitative analyses: BMD modelling to examine the effect of sampling time on mutant mutagenic potency of each tested substance

**Fig. 3:**
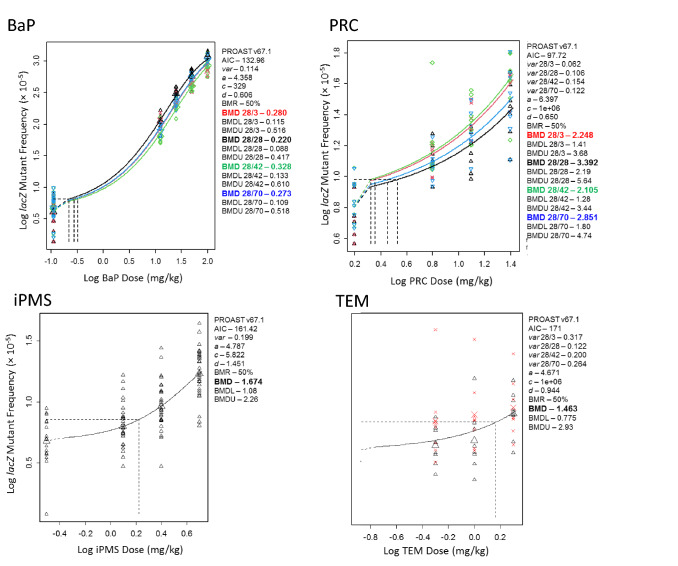
BMD Analyses of *LacZ* Mutant Frequency Dose–response Data for Each Tested Substance. PROAST output showing the relationships between Log Response (i.e., lacZ MF × 10^–5^) and Log dose (mg/kg/day) for each of the tested substances, and the fitted exponential functions. The functions were selected by minimizing AIC; in all cases the BMR was 50% (see “Materials and methods”). The horizontal dashed lines indicate the BMR, vertical dashed lines indicate the BMD(s). The text adjacent to each plot show the PROAST output indicating AIC, and estimates of parameters a, c, d, var and BMD. For each BMD, the upper and lower confidence limits are indicated as required. Small plotting symbols show results for individual animals. Where parameters are sampling time dependent, the different colours indicate the different sampling times and the BMD values are indicated using the same colours (red is 28 + 3d, black is 28 + 28d, green is 28 + 42d, and blue is 28 + 70d). *BaP* benzo(a)pyrene; *PRC* procarbazine; *iPMS* isopropyl methanesulfonate; *TEM* Triethylenemelamine (color figure online)

Next, we used the BMD combined-covariate approach to determine if the sampling time had a significant effect on mutagenic potency. For each tested chemical, the AIC value was used to select the appropriate model with which to evaluate the effect of sampling time on compound-specific BMDs. Estimates of parameters retained in models selected using the AIC did not indicate any covariate-dependence for parameters *c* (maximum response), *d* (log steepness), or *a* (background response). The most parsimonious models (i.e., the models with lowest AIC) indicated that *var* (within-group variance) was covariate dependent for PRC and TEM. Dose–response data for each compound, and the fitted exponential functions, are shown in Fig. 3 together with BMD values adjacent to each plot. For both iPMS and TEM, a single dose–response curve fitted the data for all four sampling times; thus, a single BMD value was generated (1.674 and 1.463 mg/kg for iPMS and TEM, respectively. Although the selected models for BaP and PRC produced BMD values for each sampling time (range: 0.220–0.378 and 2.195–3.392 mg/kg for BaP and PRC, respectively; Fig. 3), overlapping confidence intervals indicated that the four BMD values for each chemical were not significantly different from each other. Collectively, these analyses showed that, over the time span investigated in this study, chemical potency values (i.e., the BMDs highlighted in Fig. 3) were not significantly affected by sampling time.

## Discussion

The objective of this study was to generate critical data to evaluate the suitability of the 28 + 28d design as the only experimental protocol necessary to assess in vivo mutagenicity using OECD TG 488. Such a protocol would permit the simultaneous assessment of in vivo mutagenicity in germ cells and any somatic tissue, irrespective of the proliferation rate, thus, greatly reducing the number of animals required for testing. The results obtained in this study, which was conducted using four chemicals with greatly different fold increases in MF, strongly suggest that extending the sampling time to + 28d has no impact on assessing in vivo mutagenicity in BM. Indeed, the analysis of MF in BM at 28 + 28d provides a response that is qualitatively and quantitatively similar to that obtained with the presently recommended design of 28 + 3d.

The original decision of selecting 28 + 3d as the recommended design for somatic tissues in TG 488 was prompted by data showing a decline in MF in rapidly proliferating tissues (Hara et al. 1999; Thybaud et al. 2003) and concerns that increasing the sampling time for rapidly proliferating tissues could potentially inflate MF because of clonal expansion of mutations, especially when the response was strong (Heddle 1999; Thybaud et al. 2003). In our study, evaluation of MF over the span of 70 days from the end of exposure provided evidence for a decline in induced MF and suggested that how soon the decline manifests itself is linked to the magnitude of the effect. In the case of TEM, the mutagen that was expected to induce the smallest effect with respect to controls among the four mutagens tested, extending the sampling time past + 28d resulted in a qualitatively different outcome. In fact, while the high dose of TEM gave statistically significant increases in MF at 28 + 3d and 28 + 28d, both later sampling times failed to do so. Thus, TEM would be incorrectly classified as non-mutagenic when analyzed more than a month from the end of the exposure. Of note, sequencing of mutant plaques collected at 28 + 3d demonstrated that TEM produced a mutation spectrum that was significantly different from the spontaneous mutation spectrum further supporting its mutagenic effect (Beal et al. 2020).

Additional support for a time-related decline in MF comes from the observation that the low dose of PRC produced a statistically significant effect at all sampling times tested but 28 + 70d. Overall, these results support the notion that a decline in induced MF with increasing times from the end of the exposure is possible. A sampling time of 70 days is sufficiently long to result in the elimination from BM of the great majority of mature hematopoietic cells that had been produced while chemical exposure was taking place in both the mouse (Borghans et al. 2018) and the rat (McDonagh 1995). Thus, the decline in MF with time may reflect the differential sensitivity of long-lasting progenitor cells to the induction of mutations with respect to more advanced hematopoietic cell types. Although biologically interesting, these results have little implication for regulatory testing as it is difficult to envision a scenario under which analysis of mutations several months from the end of the exposure would be required to properly assess the hazard.

We found little evidence of increased MF with increasing sampling times as the MF for all chemicals were relatively constant at all four sampling times. MF only appeared to increase with the sampling time for the high dose of BaP which produced a > 200-fold increase in MF. Both biological and technical considerations are necessary to properly evaluate this finding. First, the MF obtained with the high dose of BaP is already extremely high at 28 + 3d approaching what would be expected with a jackpot mutation (Heddle 1999). It is possible that when the magnitude of the effect is this large, clonal expansion of mutations with time could occur to the extent that it becomes measurable. Second, the BaP results are confounded by the fact that these experiments were conducted at three different times over several years, while the other three chemicals were tested in a single experiment over a shorter period of time. Thus, there are experimental variables that could have played a role in the observed variability in MF at a high dose. Nevertheless, the BaP results are consistent, both qualitatively and quantitatively, across sampling times. Our results suggest that if there is clonal expansion of mutations with increasing sampling times, this has a negligible outcome for regulatory decision-making based on the TGR data.

Our results provide convincing evidence that observed MF in a rapidly proliferating tissue such as the BM are relatively stable over 70 days after the end of the exposure. Of primary importance for in vivo mutagenicity testing for regulatory decision-making, increasing the sampling time to + 28d for the analysis of mutations in the BM yielded qualitatively and quantitatively similar results to those obtained at + 3d. This conclusion was based on data derived from mutagens spanning two orders of magnitude in the maximum observed MF, i.e., > 200-fold versus > twofold for BaP and TEM, respectively. The results with TEM are particularly important because they show that even with a weak mutagen that barely doubles the MF with respect to controls, the 28 + 28d design is unlikely to generate false-negative results.

The results presented here were obtained in the BM, which is one of the most commonly used tissues in genotoxicity testing. It is of interest to consider how generalizable they are to other rapidly proliferating tissues, such as the small intestine that represents the site of contact for oral exposures. Despite the fact that the epithelium of the small intestine is replaced weekly, MF in the small intestine are relatively stable over 8–10 weeks post exposure regardless of whether the *lacI* (Tao et al. 1993), *lacZ* (Cosentino and Heddle 1996), *gpt* delta (Swiger et al. 2001) or the *phiX174 am3* (Cosentino et al. 2002) reporter gene is used. In all of these studies, the authors also showed that MF at the endogenous *Dbl-1* locus are also stable over this period of time. The majority of these results were obtained with single acute doses, but a similar persistence of mutations was also observed when weekly exposures were used (Tao and Heddle 1994). Together with our results, the findings in the small intestine support the notion that MF in rapidly proliferating tissues are stable for several months after the end of the exposure.

There is growing interest in applying quantitative approaches, such as BMD modelling, to analyze genotoxicity dose–response data and determine compound-specific potency (White et al. 2020). The BMD approach, which permits statistical determination of the dose that elicits a pre-defined increase in response over background (i.e., the BMR), has been deemed superior to methods that have been used to determine potency metrics such as NOGEL (no observed genotoxic effect level) and Td (threshold dose). The former is dependent on study design (i.e., dose selection and spacing) (Johnson et al. 2014), the latter requires assumptions that are generally not statistically justified. Using the BMD combined-covariate approach, the analyses conducted herein did not reveal any statistically significant impact of sampling time on compound potency (BMD). Thus, with respect to the estimation of a point of departure for quantitative interpretation of in vivo mutagenicity dose–response data, the conducted BMD analyses support the conclusion that, for rapidly proliferating tissues, the 28 + 28d design is as suitable as the 28 + 3d design.

In recent years, increased emphasis has been placed on the use of the historical background control to properly evaluate whether the results of a test are conclusively positive, negative or equivocal (Thybaud et al. 2017). Thus, an important question associated with recommending the use of 28 + 28d as the preferred design for in vivo somatic mutagenicity testing is whether it would require the establishment of a new historical background control at this time point, or whether an existing historical control at 28 + 3d would be appropriate. The analyses of MF in our set of 84 control animals indicate a trend for an age-related increase in spontaneous mutations that approaches statistical significance (e.g., *p* = 0.06). The age difference between the youngest and oldest mice in our study is ~ 100 days, and it is reasonable to assume that the age-related increase in MF would become statistically significant over a larger age span. Indeed, significant age-related increases in spontaneous mutations have been reported in several tissues using various TGR models (Aoki et al. 2015; Busuttil et al. 2007; Dolle et al. 1997, 2000; Hill et al. 2004) where the age difference between the youngest and oldest animals was larger than that in our study. These studies also demonstrated tissue-specific differences in the age-related increase in mutations; however, these differences became apparent only in much older animals than those used in our study (Aoki et al. 2015; Busuttil et al. 2007; Dolle et al. 1997, 2000; Hill et al. 2004). Importantly, our analyses showed that the age difference associated with extending the sampling time to + 28 days from + 3 days after the end of exposure is too narrow to have a significant and measurable impact on the observed MF. In fact, regardless of whether MF are analyzed by sampling time or biological age of the animals, there was no significant difference between the first two sampling times. We conclude that an existing historical control generated at 28 + 3d can be applied to new studies conducted at 28 + 28d and that there is no need to re-establish a new historical control database at the later sampling time.

In summary, we provide strong evidence that analyzing MF in rapidly proliferating tissues using the 28 + 28d design, rather than the commonly used 28 + 3d design, does not impact the ability to detect a significant increase in mutations even when a weak mutagen is employed. Furthermore, quantitative dose–response modelling of the 28 + 3d and 28 + 28d data did not reveal any statistical difference in mutagenic potency (i.e., BMD). Obviously, the 28 + 28d design requires maintaining the animals for over three additional weeks with respect to the 28 + 3d design, with associated increased maintenance costs. However, this is compensated by obtaining a more robust assessment of mutations in an important tissue for toxicological studies such as the liver, together with the possibility of using the same animals to assess MF in germ cells, which reduces by half the number of animals needed for testing. The 28 + 3d design remains an appropriate design when there is no need to obtain germ cell data or when the study is conducted in female animals for test substance-related reasons. However, following the recent adoption of the 28 + 28d design as the recommended protocol for germ cell testing (OECD 2020), our results provide robust support for selecting the 28 + 28d design as the recommended protocol in TG 488, and simultaneously assessing mutations in somatic tissues and germ cells of both mice and rats. These results provide critical information for optimizing OECD TG 488 to greatly reduce the number of animals required for regulatory testing.

## Data Availability

Individual animal mutant frequency data available upon request.

## Code availability

PROAST in R (version 67.1, https://rivm.nl/en/proast).
